# Understanding and caring for an operating microscope

**Published:** 2014

**Authors:** Ismael Cordero

**Affiliations:** Clinical Engineer, Philadelphia, Pennsylvania, USA. ismaelcordero@me.com

**Figure:**
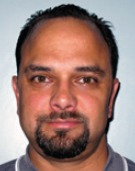
Ismael Cordero

An operating or surgical microscope is an optical instrument that provides the surgeon with a stereoscopic, high quality magnified and illuminated image of the small structures in the surgical area.

The optical components of a basic stereo microscope consist of the binocular head, a magnification changer, the objective lens and an illuminator which beams light through the objective lens and onto the operating field (Figures 1 and 2). The binocular head consists of two telescopes with adjustable eyepieces for users with refractive error. The magnification can be changed by turning a knob (which selects different magnification lenses) or by using a motorised zoom controlled by a foot pedal.

The working distance (Figure 1) is the distance from the microscope objective lens to the point of focus of the optical system. This value is fixed and is dependent on the chosen focal length of the objective lens. The choice of working distance depends on the type of surgery. For modern ophthalmic surgery that involves delicate work in the posterior chamber, objective focal lengths of 150 mm, 175 mm and 200 mm are commonly used.

The optical system often includes a beam splitter and a second set of teaching binoculars (Figure 2) so that two people can view the operation simultaneously.

The optical system is attached to the suspension arm of the floor stand (Figure 3). The suspension arm makes it possible to position the optics exactly and to fix them in place. The floor stand has wheels and can be moved around the floor and fixed into place using the brakes.

A foot pedal connected to the floor stand allows the surgeon to control the focus, the zoom, the position of the optics over the eye (the x,y position on the horizontal plane) and to turn the illumination on and off.

The illumination system is usually housed in the floor stand in order to keep the bulb heat away from the operating field. In this case, the light is transmitted to the operating field by means of a fibre optic cable. The light in ophthalmic micro scopes is usually coaxial, meaning that it follows the same path as the image in order to avoid shadows.

**Figure:**
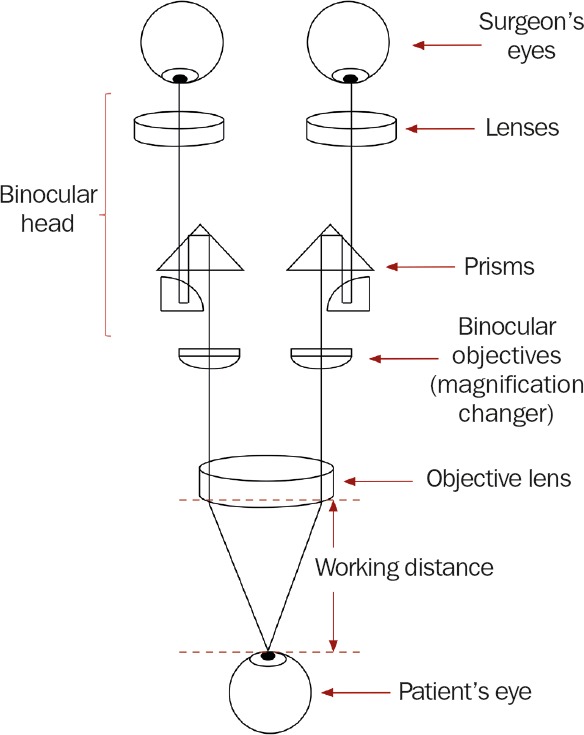
Figure 1

**Figure:**
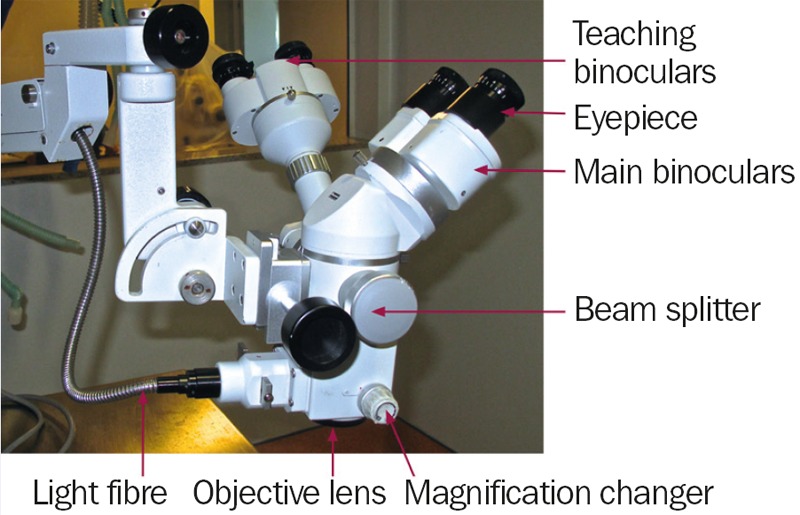
Figure 2

**Figure:**
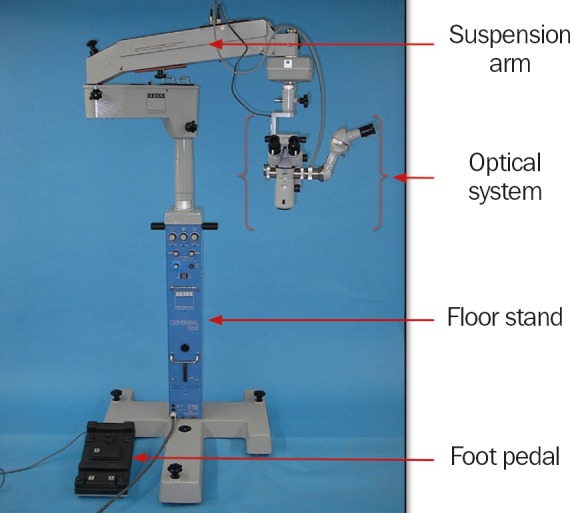
Figure 3

It is essential that all eye units develop protocols for performing microscope checks. Microscope optics should be inspected and cleaned on a weekly basis, or earlier if dirty. The entire microscope should be checked by a biomedical equipment technician at least once every six months.

## Caring for the operating microscope

- Keep the microscope in a dry, cool and well-ventilated place to prevent fungus growth on the optics (lenses).
- Every week, clean the optics according to the optical cleaning instructions described in a previous issue.^1^
- If fungus growth is detected, clean according to the instructions described in a previous issue.^2^
- To protect it from dust when not in use, drape a cover over the microscope. Vinyl coverings are preferred because they do not shed lint (like cloth coverings do). However, their use should be avoided in humid environments since they can trap moisture, which increases the risk of fungal growth.
- Wipe down the external surfaces with a damp cloth soaked in hot, soapy water.
- Cover the foot pedal with a clear plastic bag to prevent surgical and cleaning fluids from entering and damaging the electronics.
- Lift the foot pedal off the floor when washing the floor.
- Use a voltage stabiliser with the microscope. This will prevent sudden increases in voltage from destroying the bulbs and will ensure that the illumination provided remains constant.
- Before using, test the controls of the foot pedal (the x,y movement, zoom, focus, light on and off).
- Before using, check that the suspension arm can be fixed into position to ensure that it does not fall on the patient.
- Avoid kinking or bending the fibre optic cables.
- When replacing the bulbs, avoid touching them with your fingers. The oil left as fingerprints on the bulb can shorten its life.
- Do not move the microscope while the bulb is still hot because strong vibrations may damage the filament.
- Every six months, clean and oil the wheels and the brakes. Remove any surplus oil when done.
